# Risk and protection factors for self-reported hypertension and diabetes in João Pessoa, Brazil. The VIGITEL survey, 2014. A cross-sectional study

**DOI:** 10.1590/1516-3180.2017.0044250517

**Published:** 2017-08-21

**Authors:** Ana Paula Leite Moreira, Deborah Carvalho Malta, Rodrigo Pinheiro de Toledo Vianna, Patrícia Vasconcelos Leitão Moreira, Alice Teles de Carvalho

**Affiliations:** I MSc. Nutritionist, Universidade Federal da Paraíba (UFPB), João Pessoa (PB), Brazil.; II PhD. Associate Professor, Nursing School, Universidade Federal de Minas Gerais (UFMG), Minas Gerais (MG), Brazil.; III PhD. Associate Professor, Department of Nutrition, Universidade Federal da Paraíba (UFPB), João Pessoa (PB), Brazil.

**Keywords:** Chronic disease, Hypertension, Diabetes mellitus, Risk factors, Cross-sectional studies

## Abstract

**CONTEXT AND OBJECTIVE:**

Chronic diseases are the main cause of death among adults and are responsible for most outpatient and hospital care expenses in Brazil. The objective here was to determine the prevalence of hypertension and diabetes and to analyze the associations with risk and protection factors among adults.

**DESIGN AND LOCAL:**

Cross-sectional study in a state capital in northeastern Brazil.

**METHODS:**

Data on adults of both sexes aged ≥ 45 years who were interviewed in the Vigitel telephone survey in 2014 were analyzed. Prevalence ratios were estimated using Poisson regression, to identify associated factors.

**RESULTS:**

Among women, the prevalence of hypertension was 48.4% and of diabetes, 12.7%; among men, the prevalences were 41.9% and 13.8%, respectively. Multivariate analysis showed that for women, age group ≥ 65 years, overweight, self-assessed poor health and dyslipidemia remained associated with higher prevalence of hypertension. For men, overweight and self-assessed poor health remained associated with higher prevalence of hypertension. Regarding diabetes, in the multivariate model for women, age group 55-64 years, schooling level between zero and four years and no regular consumption of beans remained associated with higher prevalence. For men, age groups 55-64 years and ≥ 65 years and being married or in a stable partnership were associated with higher prevalence of diabetes.

**CONCLUSIONS:**

The results indicated that the prevalences of hypertension and diabetes were high and that preventable factors were associated with this situation, thus providing support for public policies aimed towards coping with this.

## INTRODUCTION

The four major noncommunicable diseases are cardiovascular diseases, diabetes, neoplasms and chronic respiratory diseases. These diseases have several common risk factors, which can be classified as modifiable and non-modifiable. The modifiable risk factors include smoking, abusive consumption of alcoholic beverages, excess body weight, unhealthy eating habits, sedentary lifestyle and metabolic abnormalities such as dyslipidemias. The non-modifiable risk factors are heredity, race, sex and age.^1^ The Global Burden of Disease (GBD)^2^ study, coordinated by the Institute of Metrics and Health Assessment (IHME) of the University of Washington (United States) showed that in Brazil, between 1990 and 2010, there were changes in the rankings among the ten leading causes of death. Among these causes of years of life lost due to premature death (YLLs), diabetes and hypertension increased by more than 40% over this period. The five leading causes of years of life lost due to death or disability (DALYs) among women were depression, ischemic heart disease, low back pain, cerebrovascular disease and diabetes; and among men, homicide, ischemic heart disease, car accidents, low back pain and cerebrovascular disease. Also according to this study,^2^ the risk factors that most contributed towards premature death and loss of health among men and women in Brazil in 2010 were inadequate diet, high blood pressure, overweight and altered fasting glycemia.

In the northeastern region of Brazil, data from 2014 on the major groups of causes of death that were reported by the Department of Informatics of the Brazilian National Health System (DATASUS)^3^ showed that diseases of the circulatory and endocrine systems and nutritional and metabolic diseases corresponded, respectively, to proportions of deaths of 27.56% and 7.52%, in comparison with the total number of deaths from all causes. In João Pessoa, the state capital of Paraíba, which was the subject of the present study, data from 2014 also reported by DATASUS^3^ showed that diseases of the circulatory and endocrine systems and nutritional and metabolic diseases corresponded, respectively, to proportions of deaths of 27.48% and 6.67%, in comparison with the total number of deaths from all causes.

Diabetes is a highly incapacitating disease that can cause diabetic retinopathy, amputations, nephropathies, cardiovascular and encephalic complications, among other conditions. It can impair individuals’ functional capacity, autonomy and quality of life, thus resulting in high social and financial costs for society and for these individuals and their families.^4^

Hypertension is considered to be both a chronic disease and a risk factor for other diseases and chronic conditions, such as chronic kidney disease and diabetes, among others. This gives it greater prominence as these individuals’ health conditions worsen, thus contributing towards loss of quality of life, early lethality of diseases and high costs for social and healthcare systems. It has a multifactorial nature, with an asymptomatic course in many cases, which means that this diagnosis is neglected and, consequently, so is treatment. In addition, hypertension is highly prevalent in Brazil and in the world, thus representing a great challenge for public health.^5^^,^^6^

A study carried out on the adult population of Campinas,^7^ state of São Paulo, showed that there were significant differences in the prevalences of risk and protection factors for chronic diseases according to gender. The prevalences of smoking, former smokers, alcohol abuse, overweight, obesity and free-time physical activity were higher among men; among women, healthier eating habits and dyslipidemia were more prevalent.

Chronic diseases are responsible for the greatest proportion of the burden of diseases diagnosed in Brazil and present significant modifiable risk factors. The impact of these diseases and their risk factors varies according to gender and the level of development of the different regions of the country. Moreover, chronic diseases are highly prevalent among people aged 45 years and over.

Because very few studies on hypertension and diabetes have been conducted in João Pessoa, the aims of the present study were to ascertain the prevalence of these diseases and to identify and measure the independent effects of risk and protection factors relating to the presence of previous medical diagnoses of these diseases, as reported by adults in this municipality.

## OBJECTIVE

The objectives of this study were to ascertain the prevalence of hypertension and diabetes and to identify the relationships of sociodemographic and behavioral characteristics, food consumption characteristics and health indicators towards the presence of previous medical diagnoses of these two chronic diseases, as reported by adults, stratified according to sex, in a state capital in northeastern Brazil.

## METHODS

This was a cross-sectional, population-based, epidemiological study that used data from the Surveillance of Risk and Protection Factors for Chronic Diseases by Telephone (Vigitel) survey. The project for implementing Vigitel was approved by the National Ethics Committee for Research on Human Beings (CONEP) of the National Health Council (CNS), Ministry of Health, under report no. 355.590, of June 26, 2013, and under the certificate of presentation for ethics assessment (CAAE) no. 16202813.2.0000.0008. Since the project related to telephone interviews, the free and informed consent document was replaced by verbal consent that was obtained by the Ministry of Health at the time of the interview. To conduct the present study, the coordination office for non-transmissible diseases and health hazards of the Secretariat for Health Surveillance, Department of Health Situation Analysis, Ministry of Health, made data from Vigitel 2014 relating to João Pessoa (capital of the state of Paraíba) available to us.

Adults aged 45 years and over who were living in households in João Pessoa served by at least one landline telephone in the year 2014 were included. During that year, 1,517 adults aged 18 and over living in this city were interviewed as part of the Vigitel survey.^8^^,^^9^ Out of this total, 867 interviews were conducted with adults aged 45 years and over, i.e. the target audience of the present study. These individuals comprised 566 females (65.28%) and 301 males (34.71%). This sample of 867 adults was weighted according to sex, age and schooling, in accordance with the methodology established for Vigitel, using the “rake” method,^8^^,^^9^^,^^10^^,^^11^thus making the data of this sample representative of the total adult population of this capital. Details of the sampling process and the weighting of Vigitel estimates, along with other details of the methodology used by this system can be seen in other published papers.^8^^,^^9^ The electronic questionnaire used at the time of the interviews is available in the annual publication of results from Vigitel.^9^

The dependent variables analyzed in this study were the prevalence of hypertension and the prevalence of diabetes, as reported by adults who had previously received these medical diagnoses. They were defined by the percentages of adults who reported having a prior medical diagnosis as positive answers to the following questions, respectively: “Has any doctor ever told you that you have high blood pressure?”; and “Has any doctor ever told you that you have diabetes?”.

The independent variables analyzed in this study were selected based on their importance for determining the total burden of disease, as estimated by the World Health Organization for the Americas region.^12^ They consisted of risk and protection indicators selected from Vigitel, and were grouped into: sociodemographic, behavioral, food consumption and health indicators. The sociodemographic variables were: age group (45-54, 55-64 or ≥ 65 years); marital status (single, married/stable partnership, widowed or separated/divorced); schooling level (0-4, 5-8, 9-11 or ≥ 12 years of study); and possession of health insurance (yes or no). The behavioral categories included: smokers (adults who reported being current smokers, regardless of the number of cigarettes, frequency and duration of smoking); former smokers (adults who reported being former smokers, regardless of time elapsed); and physically inactive individuals (adults who had not exercised during their free time within the last three months, were not performing any intense physical efforts at work, were not going to work/school on foot or by bicycle with a minimum journey time of 20 minutes and were not performing heavy cleaning in their homes). The food consumption variables were: regular consumption of fruits, vegetables and greens (on five or more days of the week); recommended consumption of fruits, vegetables and greens (five servings daily, on five or more days of the week); regular consumption of beans (on five or more days of the week); consumption of meat with excess fat (habit of consuming red meat with visible fat and/or chicken with skin); consumption of milk with full fat content (habit of consuming whole milk fat); regular consumption of soda or artificial juice (on five or more days of the week); regular consumption of sweets (on five or more days per week); replacement of lunch or dinner by snacks (seven or more times a week); and high salt intake (adults who considered that their salt intake was high or too high). The health indicators analyzed the following conditions: overweight, in terms of the body mass index (BMI); defined as body weight (kg) divided by square of height (m^2^), for which self-reported information on weight and height was used to calculate BMI and adults who presented BMI ≥ 25 kg/m^2^ were considered to be overweight, as classified by the World Health Organization;^13^ self-assessed poor/very poor health (adults who assessed their health as poor or very poor, in answer to the question “Would you rate your health as: very good, good, normal, poor, or very poor?”); and dyslipidemia (adults who reported having a previous medical diagnosis of dyslipidemia: high cholesterol or triglycerides).

All the analyses were performed using the Vigitel expansion factor, using the Stata survey procedure version 11 SE. Initially, Pearson chi-square association tests were performed to verify the existence of a statistical association between the independent variables and outcomes (P ≤ 0.05). Subsequently, the Poisson regression model was used to verify the existence of factors associated with arterial hypertension and diabetes. The variables that presented P ≤ 0.20 in the univariate analysis were considered for introduction into the multivariate model. The magnitudes of the associations found were measured using prevalence ratios (PR) with their respective 95% confidence intervals (95% CI).

## RESULTS

Data from 867 adults aged 45 years and over were analyzed. These comprised 566 women and 301 men, corresponding to 65.28% and 34.72% of the total sample, respectively. Another 271 adults were excluded because they were 18-44 years old. Most of these individuals were between 25-34 years old, had 9 to 11 years of educational attainment and had no health insurance.

Previous medical diagnoses of hypertension and diabetes were reported by 45.8% and 13.1% of the population, respectively, and there were no significant differences between the genders.

Table 1 describes the sociodemographic, behavioral, food consumption and health indicators of the study population and the presence of hypertension and diabetes, stratified according to gender. Among the women, there was higher prevalence of hypertension in the age group ≥ 65 years (95% CI: 50.6-66.2) and among those who did not consume whole milk (95% CI: 46.8-59.9); those who were overweight (95% CI: 52.2-65.4); those who self-rated their health as poor/very poor (95% CI: 48.1-85.2); and those who reported having a medical diagnosis of dyslipidemia (95% CI: 55.6-71.3). Among the men, there was statistically significant higher prevalence of hypertension in the age group ≥ 65 years (95% CI: 39.1-62.2) and among those who reported consuming meats with excessive fat (95% CI: 17.6-43.6); those who were overweight (95% CI: 39.0-56.6); those who self-assessed their health as poor (95% CI: 64.2-97.6); and those who reported having a medical diagnosis of dyslipidemia (95% CI: 42.0-67.4).

**Table 1:** Prevalences of hypertension and diabetes in the population, according to gender and sociodemographic, behavioral, food consumption and health indicator characteristics. João Pessoa, Paraíba, Brazil (2014)

Variables	Hypertension				Diabetes			
	Female sex		Male sex		Total P (%)*	Female sex		Male sex		Total P (%)*
	P (%)*	P-value^**†**^	P (%)*	P-value^**†**^		P (%)*	P-value^**†**^	P (%)*	P-value^**†**^	
Sociodemographic indicators										
Age group										
45-54 years	40.1	0.010	31.9	0.038	36.7	6.8	0.011	4.3	0.001	5.8
55-64 years	51.5		50.0		50.9	17.3		24.4		20.2
≥ 65 years	58.6		50.7		55.6	17.2		18.5		17.7
Marital status										
Single	38.7	0.278	36.9	0.892	38.1	9.2	0.401	0.6	0.000	6.3
Married/stable partnership	48.6		42.7		45.7	12.1		17.4		14.7
Widowed	49.9		49.7		49.9	18.1		4.6		16.9
Separated/divorced	57.5		39.1		51.0	15.5		3.8		11.4
Education										
≥ 12 years	42.2	0.298	35.0	0.544	39.4	5.8	0.048	15.1	0.889	9.3
9-11 years	45.4		46.5		45.8	10.2		13.2		11.3
5-8 years	48.6		36.4		44.0	14.0		11.3		13.0
0-4 years	56.2		45.3		51.2	19.1		15.6		17.5
Possession of health insurance										
Yes	45.5	0.337	49.5	0.105	47.0	10.4	0.277	16.7	0.387	12.7
No	50.5		37.7		45.2	14.0		12.2		13.2
Behavioral indicators										
Smokers										
No	49.4	0.092	42.0	0.950	46.7	12.4	0.414	13.3	0.686	12.7
Yes	31.2		41.4		38.1	18.4		16.2		16.9
Former smokers										
No	48.7	0.875	42.4	0.870	46.4	14.1	0.197	9.8	0.046	12.5
Yes	47.8		41.2		44.7	9.2		20.2		14.4
Physically inactive										
No	46.2	0.129	38.4	0.117	43.1	11.2	0.130	13.8	0.988	12.2
Yes	55.4		51.7		53.8	12.7		13.9		15.8
Food consumption indicators										
Regular consumption of FVG										
Yes	45.5	0.265	43.6	0.687	44.8	11.9	0.624	13.7	0.981	12.5
No	51.4		40.7		46.7	13.5		13.9		13.7
Recommended consumption of FVG										
Yes	47.2	0.778	45.7	0.571	46.7	11.0	0.512	10.5	0.398	10.8
No	48.8		40.8		45.5	13.3		14.8		13.9
Regular consumption of beans										
Yes	47.4	0.527	41.2	0.572	44.6	10.6	0.049	13.9	0.880	12.1
No	50.9		46.4		49.7	18.0		12.9		16.7
Meat with excessive fat										
No	49.3	0.323	46.1	0.041	48.1	12.0	0.259	13.4	0.835	12.5
Yes	40.4		28.9		33.2	18.7		14.9		16.3
Milk with full fat content										
No	53.4	0.024	41.5	0.875	48.6	15.2	0.077	15.8	0.320	15.4
Yes	41.0		42.6		41.6	9.0		10.9		9.8
Regular consumption of soda or artificial juice										
No	48.6	0.746	43.7	0.183	46.7	12.5	0.609	14.4	0.452	13.2
Yes	44.9		27.5		34.5	17.0		9.0		12.2
Regular consumption of sweets										
No	47.9	0.568	43.5	0.099	46.1	13.6	0.087	14.5	0.209	14.0
Yes	51.9		26.5		43.5	6.3		6.5		6.4
Replacement of lunch or dinner with snack										
No	49.9	0.083	42.9	0.284	45.3	1.6	0.032	14.4	0.163	13.9
Yes	61.3		29.1		51.0	4.7		5.9		5.1
High salt consumption										
No	47.6	0.113	42.7	0.571	45.7	12.7	0.916	14.7	0.429	13.5
Yes	64.3		36.1		47.0	12.0		7.1		9.0
Health indicators										
BMI - overweight										
No	32.4	0.000	28.4	0.013	31.1	8.6	0.042	6.1	0.009	7.7
Yes	59.0		47.7		54.0	15.4		17.1		16.1
Self-assessed poor/very poor health										
No	47.0	0.039	39.2	0.000	43.8	12.7	0.887	12.9	0.125	12.8
Yes	69.8		89.6		77.1	11.8		28.7		18.0
Self-reported dyslipidemia										
No	38.2	0.000	36.1	0.017	37.3	48.9	0.092	13.2	0.702	11.6
Yes	63.8		55.0		60.8	51.1		15.2		15.8

Note: the results express the percentage for the population. *Prevalence; ^†^Statistical analysis performed was Pearson’s chi-square test. BMI = body mass index; FVG = fruits, vegetables and greens.

There was higher prevalence of diabetes among women over 55 years old (95% CI: 11.0-26.2); among those with 0-4 years of education (95% CI: 11.8-29.5); among those who did not consume beans regularly (95% CI: 11.9-26.2); among those who did not replace lunch or dinner with snacks (95% CI: 10.3-17.7); and among those who were overweight (95% CI: 11.2-20.8). There was higher prevalence of diabetes among men in the age group of 55-64 years (95% CI: 13.5-39.9); among those who were married or were in a stable partnership (95% CI: 11.8-24.8); among those who reported being former smokers (95% CI: 12.0-32.1); and among those who were overweight (95% CI: 11.3-25.1) (Table 1).

Table 2 shows the crude and adjusted analyses on factors associated with hypertension in women and men. The adjusted analysis relating to the women’s data showed that the age group ≥ 65 years old, excessive body weight, self-rated poor health and a previous medical diagnosis of dyslipidemia remained independently associated with higher prevalence of hypertension. Consuming whole-fat milk remained associated with lower prevalence of hypertension in women. In the adjusted analysis for men, overweight and self-rated poor health were independently associated with hypertension. The results differed between the age groups of 55-64 years and ≥ 65 years. Presence of a previous medical diagnosis of dyslipidemia lost its statistical significance in the adjusted analysis.

**Table 2:** Prevalence and prevalence ratios (crude and adjusted) for prior medical diagnosis of hypertension reported by women (n = 566) and men (n = 300), according to sociodemographic, behavioral, food consumption and health indicator variables. João Pessoa, Paraíba, Brazil (2014)

Variables	Women		Men	
	Crude PR* 95% CI*	Adjusted^**†**^ PR* 95% CI*	Crude PR* 95% CI*	Adjusted^**†**^ PR* 95% CI*
Sociodemographic indicators				
Age group				
45-54 years	1.0	1.0	1.0	1.0
55-64 years	1.2 (0.9-1.7)^‡^	1.1 (0.9-1.5)	1.5 (1.0-2.4)^‡^	1.3 (0.9-2.0)
≥ 65 ears	1.4 (1.1-1.8)^‡^	1.3 (1.0-1.7)^§^	1.5 (1.0-2.3)^‡^	1.3 (0.9-1.9)
Marital status				
Single	1.0	-	1.0	-
Married/stable partnership	1.2 (0.8-1.8)	-	1.1 (0.5-2.2)	-
Widowed	1.2 (0.8-1.8)	-	1.3 (0.5-3.3)	-
Separated/divorced	1.4 (0.9-2.2)	-	1.0 (0.4-2.7)	-
Education				
≥ 12 years	1.0	-	1.0	-
9-11 years	1.0 (0.8-1.4)	-	1.3 (0.8-1.9)	-
5-8 years	1.1 (0.8-1.5)	-	1.0 (0.6-1.7)	-
0-4 years	1.3 (0.9-1.7)	-	1.2 (0.8-2.0)	-
Possession of health insurance				
Yes	1.0	-	1.0	1
No	1.1 (0.8-1.3)	-	0.7 (0.5-1.0)^‡^	0.8 (0.6-1.1)
Behavioral indicators				
Smokers				
No	1.0	1.0	1.0	-
Yes	0.6 (0.3-1.1)^‡^	0.6 (0.4-1.1)	0.9 (0.6-1.6)	-
Former smokers				
No	1.0	-	1.0	-
Yes	0.9 (0.7-1.2)	-	0.9 (0.6-1.3)	-
Physically inactive				
No	1.0	1.0	1.0	1.0
Yes	1.1 (0.9-1.5)^‡^	0.9 (0.7-1.2)	1.3 (0.9-1.9)^‡^	1.1 (0.8-1.6)
Food consumption indicators				
Regular consumption of FVG				
Yes	1.0	-	1.0	-
No	1.1 (0.9-1.4)	-	0.9 (0.6-1.3)	-
Recommended consumption of FVG				
Yes	1.0	-	1.0	-
No	1.0 (0.8-1.3)	-	0.8 (0.6-1.3)	-
Regular consumption of beans				
Yes	1.0	-	1.0	-
No	1.0 (0.8-1.3)	-	1.1 (0.7-1.6)	-
Meats with excessive fat				
No	1.0	-	1.0	1.0
Yes	0.8 (0.5-1.2)	-	0.6 (0.3-1.0)^‡^	0.7 (0.4-1.2)
Milk with full fat content				
No	1.0	1.0	1.0	-
Yes	0.7 (0.6-0.9)^‡^	0.7 (0.6-0.9)^§^	1.0 (0.7-1.4)	-
Regular consumption of soda or artificial juice				
No	1.0	-	1.0	1.0
Yes	0.9 (0.5-1.5)	-	0.6 (0.2-1.3)	0.7 (0.4-1.4)
Regular consumption of sweets				
No	1.0	-	1.0	1.0
Yes	1.0 (0.8-1.4)	-	0.6 (0.3-1.1)^‡^	0.7 (0.3-1.3)
Replacement of lunch or dinner with snack				
No	1.0	1.0	1.0	-
Yes	1.3 (0.9-1.7)^‡^	1.2 (0.9-1.5)	0.6 (0.3-1.4)	-
High salt intake				
No	1.0	1.0	1.0	-
Yes	1.3 (0.9-1.8)^‡^	1.2 (0.9-1.7)	0.8 (0.4-1.5)	-
Health indicators				
BMI - overweight				
No	1.0	1.0	1.0	1.0
Yes	1.8 (1.4-2.3)^‡^	1.7 (1.3-2.2)^§^	1.6 (1.0-2.6)^‡^	1.7 (1.1-2.5)^§^
Self-assessed poor/very poor health				
No	1.0	1.0	1.0	1.0
Yes	1.4 (1.1-2.0)^‡^	1.3 (1.0-1.8)^§^	2.2 (1.7-2.9)^‡^	1.9 (1.4-2.5)^§^
Dyslipidemia				
No	1.0	1.0	1.0	1.0
Yes	1.6 (1.3-2.0)^‡^	1.5 (1.2-1.8)^§^	1.5 (1.0-2.1)^‡^	1.2 (0.9-1.7)

*PR = prevalence ratio; CI = confidence interval; ^†^Statistical analysis adjusted using Poisson regression, performed only on independent variables that presented significance ≤ 0.20 (P ≤ 0.20) in Pearson’s chi-square test; ^‡^P ≤ 0.20; ^§^P ≤ 0.05. BMI = body mass index; FVG = fruits, vegetables and greens.

Table 3 shows the crude and adjusted analyses on factors associated with diabetes in women and men. In the adjusted analysis relating to women, the age group of 55-64 years, educational level of 0-4 years and not consuming beans regularly remained independently associated with higher prevalence of diabetes. This differed from the age group ≥ 65 years and educational attainment of 5-8 years, which lost their association with the prevalence of diabetes in the adjusted analysis. Replacing meals with snacks remained associated with lower prevalence of diabetes in women. In the adjusted analysis for men, the age groups of 55-64 years and ≥ 65 years remained independently associated with diabetes, along with being married or in a stable partnership.

**Table 3:** Prevalence and prevalence ratios (crude and adjusted) for prior medical diagnosis of diabetes reported by women (n = 566) and men (n = 297), according to sociodemographic, behavioral, food consumption and health indicator variables. João Pessoa, Paraíba, Brazil (2014)

Variables	Women		Men	
	Crude PR* 95% CI*	Adjusted^**†**^ PR* 95% CI*	Crude PR* 95% CI*	Adjusted^**†**^ PR* 95% CI*
Sociodemographic indicators				
Age group				
45-54 years	1.0	1.0	1.0	1.0
55-64 years	2.5 (1.1-5.4)^‡^	2.0 (0.9-4.4)^§^	5.6 (2.1-14.8)^‡^	5.1 (1.9-13.4)^§^
≥ 65 years	2.5 (1.2-5.1)^‡^	1.8 (0.8-3.9)	4.2 (1.6-10.7)^‡^	4.0 (1.4-10.9)^§^
Marital status				
Single	1.0	-	1.0	1.0
Married/stable partnership	1.3 (0.5-3.1)	-	27.8 (3.5-216.9)^‡^	17.7 (2.0-153.0)^§^
Widowed	1.9 (0.8-4.7)	-	7.4 (0.6-91.4)^‡^	3.7 (0.2-54.9)
Separated/divorced	1.6 (0.5-5.3)	-	6.1 (0.4-77.2)^‡^	6.5 (0.4-97.3)
Education				
≥ 12 years	1.0	1.0	1.0	-
9-11 years	1.7 (0.7-4.0)	1.7 (0.7-4.1)	0.8 (0.4-1.8)	-
5-8 years	2.4 (0.9-5.8)	2.0 (0.8-5.0)	0.7 (0.2-2.1)	-
0-4 years	3.3 (1.4-7.7)^‡^	2.5 (1.0-6.3)^§^	1.0 (1.4-2.5)	-
Possession of health insurance				
Yes	1.0	-	1.0	-
No	1.3 (0.7-2.3)	-	0.7 (0.3-1.4)	-
Behavioral indicators				
Smokers				
No	1.0	-	1.0	-
Yes	1.4 (0.5-3.7)	-	1.2 (0.4-3.1)	-
Former smokers				
No	1.0	1.0	1.0	1.0
Yes	0.6 (0.3-1.2)^‡^	0.7 (0.3-1.4)	2.0 (1.0-4.2)^‡^	1.6 (0.8-3.2)
Physically inactive				
No	1.0	1.0	1.0	-
Yes	1.5 (0.8-2.6)	1.0 (0.6-1.7)	1.0 (0.4-2.2)	-
Food consumption indicators				
Regular consumption of FVG				
Yes	1.0	-	1.0	-
No	1.1 (0.6-1.9)	-	1.0 (0.4-2.1)	-
Recommended consumption of FVG				
Yes	1.0	-	1.0	-
No	1.2 (0.6-2.1)	-	1.4 (0.6-3.1)	-
Regular consumption of beans				
Yes	1.0	1.0	1.0	-
No	1.6 (1.0-2.8)^‡^	1.6 (1.0-2.7)^§^	0.9 (0.3-2.5)	-
Meats with excessive fat				
No	1.0	-	1.0	-
Yes	1.5 (0.7-3.2)	-	1.1 (0.4-2.8)	-
Milk with full fat content				
No	1.0	1.0	1.0	-
Yes	0.5 (0.3-1.0)^‡^	0.6 (0.3-1.1)	0.6 (0.3-1.4)	-
Regular consumption of soda or artificial juice				
No	1.0	-	1.0	-
Yes	1.3 (0.4-4.4)	-	0.6 (0.1-2.2)	-
Regular consumption of sweets				
No	1.0	1.0	1.0	1.0
Yes	0.4 (0.1-1.1)^‡^	0.5 (0.1-1.3)	0.4 (0.1-1.7)^‡^	0.5 (0.1-2.1)
Replacement of lunch or dinner with snack				
No	1.0	1.0	1.0	1.0
Yes	0.3 (0.1-0.9)^‡^	0.3 (0.1-1.0)^§^	0.4 (0.1-1.5)^‡^	0.6 (0.1-2.6)
High salt intake				
No	1.0	-	1.0	-
Yes	0.9 (0.3-2.7)	-	0.4 (0.7-3.2)	-
Health indicators				
BMI - overweight				
No	1.0	1.0	1.0	1.0
Yes	1.7 (1.0-3.1)^‡^	1.7 (0.9-3.0)	2.8 (1.2-6.3)^‡^	2.2 (0.9-4.9)
Self-assessed poor/very poor health				
No	1.0	-	1.0	1.0
Yes	0.9 (0.3-2.6)	-	2.2 (0.8-5.8)^‡^	1.8 (0.7-4.4)
Dyslipidemia				
No	1	1	1.0	-
Yes	1.5 (0.9-2.6)^‡^	1.3 (0.8-2.1)	1.1 (0.5-2.3)	-

*PR = prevalence ratio; CI = confidence interval; ^†^Statistical analysis adjusted using the Poisson regression, performed only on independent variables that presented significance ≤ 0.20 (P ≤ 0.20) in Pearson’s chi-square test; ^‡^P ≤ 0.20; ^§^P ≤ 0.05. BMI = body mass index; FVG = fruits, vegetables and greens.

## DISCUSSION

The results from the present study identified factors associated with hypertension and diabetes in the study population. For women, hypertension remained associated with the age group ≥ 65 years old and with higher prevalence of overweight, self-rated poor/very poor health and dyslipidemia. For men, hypertension was associated with higher prevalence of overweight and self-rated poor/very poor health. Regarding diabetes, in women, the age group of 55-64 years, educational attainment of 0-4 years and regular non-consumption of beans were associated with higher prevalence of this chronic disease. In men, the age groups of 55-64 years and ≥ 65 years were associated with higher prevalence of diabetes, along with being married or in a stable partnership.

The prevalence of hypertension identified in the present study was greater than that found through the Vigitel survey in João Pessoa (the state capital of Paraiba) in the years 2012^14^ and 2013,^15^ when the self-reported frequencies of a medical diagnosis of this chronic disease among adults aged ≥ 18 years were 25.7% and 24.4%, respectively. The prevalence of diabetes was similar to that found in a study conducted in Florianópolis,^16^ Santa Catarina, Brazil, in which data on elderly people aged ≥ 60 years with self-reported diagnoses of diabetes were evaluated. Moreover, the prevalence of diabetes identified in the present study was also greater than that found through the Vigitel survey in João Pessoa in the years 2012^14^ and 2013,^15^ when the self-reported frequencies of a medical diagnosis of diabetes among adults were 5.9% and 6.5%, respectively.

In the present study, higher prevalences of hypertension and diabetes occurred with advancing age, in agreement with previous research. Higher prevalence of noncommunicable diseases with advancing age is an expected result because of the characteristics of these diseases and structural and physiological changes that occur in the body during aging.^16^^,^^17^ However, it is worth mentioning that, regarding diabetes, there was a slight decrease in prevalence with advancing age. This inverse relationship was also found in the ISA-SP^18^ project (Health Surveys in the State of São Paulo) and in the SABE^19^ study (Health, well-being and aging). One possible explanation might relate to survival bias, given the greater mortality among diabetics with increasing age, due to the great number of complications resulting from this disease.^4^^,^^18^

Self-assessed poor/very poor health was associated with hypertension in both genders. The literature generally indicates that health evaluations are worse among women, since they are the individuals who access healthcare services the most. Thus, women have greater concern for and perception of their health. On the other hand, men tend to self-evaluate their health as poor only in the presence of some disease.^16^ In a study carried out by Carvalho et al.,^20^ the prevalence of self-assessed poor health was significantly higher among individuals with lower educational level, those with chronic disease (hypertension, diabetes or obesity) and women, both in northeastern Brazil and in Portugal.

The present study also showed that dyslipidemia was significantly associated with hypertension among women. There is evidence of a correlation between lipid profile and systemic arterial pressure, as observed in metabolic syndrome.^21^ Regarding food consumption variables, despite the consensus in the literature that consumption of foods that are considered to be risk factors for non-communicable chronic diseases (such as high-fat foods) and replacement of meals by snacks (usually composed of snack foods and fast food) have direct relationships with occurrences of chronic diseases, the present study did not confirm these association. These results may have two explanations: the women involved might already have been undergoing treatment, with dietary reeducation to control hypertension and diabetes, thus forming a framework of reverse causality; or these women might also have distorted their reporting of some foods, if they already knew their beneficial or even harmful effects on health. Individuals with diagnoses of non-communicable chronic diseases are more likely to attend healthcare services, where they are advised to change their eating and healthcare behaviors. In such cases, the inverse association is a positive indicator.^22^^,^^23^

Starting with the 2013 Vigitel survey,^24^ questions regarding replacement of meals by snacks were included. In João Pessoa, interviewees in this situation who had been replacing meals with regional foods such as tapioca, couscous and other items that do not fit the definition of dinner may have been included. The questionnaire only became more specific after Vigitel 2015, through inclusion of positive responses regarding consumption of pizzas, sandwiches and other processed snacks, thus excluding items that are common in some regions, such as couscous and *tacacá*, among others.

Not consuming beans regularly was associated with higher prevalence of diabetes among women. Beans are legumes that traditionally have formed part of the Brazilian diet and adequate consumption of beans has been strongly associated with protection against several diseases, since it is one of the foods with proportionally larger amounts of dietary fiber, compared with other foods and constitutes an important item within healthy food consumption.^25^ Some Brazilian studies on populations in the northeastern and southeastern regions have shown that bean consumption has beneficial effects at the population level, through providing protective effects against body weight gain.^26^

The present study also found an association between lower schooling level (0-4 years) and higher prevalence of diabetes among women. This was similar to the findings from a study carried out in the city of Viçosa, Minas Gerais,^27^ in which higher schooling levels were inversely associated with occurrences of diabetes among 621 elderly individuals aged 60 years and over. Likewise, in a study carried out in the municipality of Triunfo,^28^ in the backlands of the state of Pernambuco, Brazil, on a representative sample of 198 adults with a mean age of 57.7 years, all the cases of diabetes were among individuals who were illiterate or had only had elementary education. The fact that the higher the schooling level is, the lower the chances are that individuals will develop hypertension and/or diabetes, demonstrates that government investment in education is paramount. Low educational level can hinder access to healthcare information and limit understanding of the guidelines regarding prevention and/or treatment of diabetes.^27^

Regarding marital status, for men, being married or in a stable partnership showed a statistically significant association with occurrences of diabetes. This result was contrary to those of other studies, such as GAZEL,^29^ in which reports of non-communicable chronic diseases were more frequent among individuals living alone. That association seemed to result from greater exposure to behavioral risk factors for chronic diseases among single individuals.

The results from the present study also demonstrated that overweight was a determinant strongly associated with occurrences of hypertension. Other studies on elderly individuals in the municipalities of Marques de Souza (Rio Grande do Sul)^30^ and Bauru (São Paulo)^17^ and from the Vigitel survey,^31^ conducted in all Brazilian state capitals, showed that between 20% and 30% of the prevalence of hypertension could be explained by an association between overweight and increased risk of developing this disease.^31^

Some limitations of the present study need to be pointed out. One limitation to be highlighted relates specifically to the methodology used in the Vigitel system: only individuals living in households that have a landline can be interviewed, which gives rise to the possibility of calibration bias. However, weighting factors through which it is sought through post-stratification to estimate the prevalence taking into account differences in the demographic characteristics of the Vigitel sample in relation to those of the entire population are used. Furthermore, the high response rate achieved through Vigitel contributes to the quality of the data.

Another limitation relates to the use of self-reported data, which can be influenced by individuals’ access to medical diagnoses and their understanding of their health condition. The potential for information bias, with overestimation of height and underestimation of weight, cannot be discarded given that Vigitel provides self-reported and unmeasured weight and height. However, validation studies on some Vigitel indicators have been conducted in Brazil,^32^^,^^33^ showing agreement between the information reported through Vigitel and the information from household surveys. Vigitel has the advantage of being a non-invasive method in which it is easy to obtain data, at low cost.

Since Vigitel has a cross-sectional methodological design, it is not possible to establish any temporal cause-and-effect relationship among the associations between outcomes and independent variables. Therefore, it cannot distinguish whether the factors associated with hypertension and diabetes are causes or consequences of illness. However, recognizing the risk factors associated with chronic diseases is essential for identifying groups with specific needs and for guiding public policies, through establishing appropriate monitoring of these risk factors.

A further limitation of the present study relates to its extraction of Vigitel data, in which data from only one Brazilian state capital were separated out. This potentially reduced the statistical power of the tests through decreasing the sample size.

Moreover, only adults aged 45 and over were included, thus hindering knowledge of the behavior of the population under 45 years old. Since hypertension and diabetes are chronic diseases, these factors may have changed over the course of life, thereby reducing the effect observed in this study. However, this is a limitation of the cross-sectional study design. 

Despite the limitations identified, some potentialities of this study stood out. Cross-sectional population-based studies on representative samples conducted through telephone surveys are of great relevance because they are fast and cost-effective alternatives. They thus constitute an important epidemiological tool for determining the dimensions of problems, through estimating indicators for health conditions, health-related behaviors and access to and use of healthcare and disease treatment services. Such studies provide support for actions that may be implemented to promote health and prevent non-communicable chronic diseases in the reference population or in others with similar characteristics.

## CONCLUSION

The results obtained confirmed the importance of hypertension and diabetes as a public health problem and identified a list of factors associated with these chronic diseases, among which some would be susceptible to intervention. Thus, this study identified an urgent need for specific interventions in this population, with implementation of healthcare aimed towards minimizing the complications arising from these pathological conditions, as well as preventing the onset of other chronic diseases. These interventions should be conducted in such a way that they allow individuals to discuss issues relating to their chronic conditions and the risk factors involved, while at the same time enabling stimulation and providing conditions that encourage these individuals to adopt healthier lifestyles.
